# Delayed Rupture of Extensor Tendons of Index Finger—An Unusual Complication after Distal Radius Fracture Fixation with Intramedullary nail: A Case Report and Review of Literature

**DOI:** 10.1055/s-0045-1813258

**Published:** 2025-12-09

**Authors:** Maksud Mubarak Devale, Sarika Rao, Ritika Parmar, Nayana Bhortakay

**Affiliations:** 1 Department of Plastic Surgery, Lokmanya Tilak Municipal General Hospital, Mumbai, Maharashtra, India

**Keywords:** attrition rupture, extensor tendons, distal radius fracture

## Abstract

Delayed rupture of extensor tendons of hand can occur due to a variety of reasons. Post-traumatic attrition rupture of extensor pollicis longus (EPL) tendon following distal radius fractures is well known. We report an extremely rare case of isolated rupture of both the extensor digitorum communis (EDC) and extensor indicis proprius (EIP) tendons of index finger due to intramedullary nailing without EPL rupture.

## Introduction

Delayed, spontaneous rupture of extensor tendons of hand can occur due to a variety of reasons. Common in hands with advanced rheumatoid arthritis, late rupture can also occur after fractures of distal radius, carpal, or metacarpal bones. Post-traumatic attrition rupture of extensor pollicis longus (EPL) tendon following distal radius fractures or its fixation is well known. We report an extremely rare case of isolated rupture of both the extensor digitorum communis (EDC) and extensor indicis proprius (EIP) tendons of index finger due to intramedullary nailing without EPL rupture.

## Case Presentation

A right-handed, 25-year-old male, a fire alarm repair technician, presented to us with inability to extend his right index finger since 3 months after a trivial, blunt trauma to the dorsum of his distal forearm. He had sustained distal radius fracture in the same limb 2.5 years ago due to an accidental fall. The fracture was fixed with intramedullary titanium elastic nail system (TENS). He had regained full wrist and finger motion after that surgery.

**Fig. 1:**
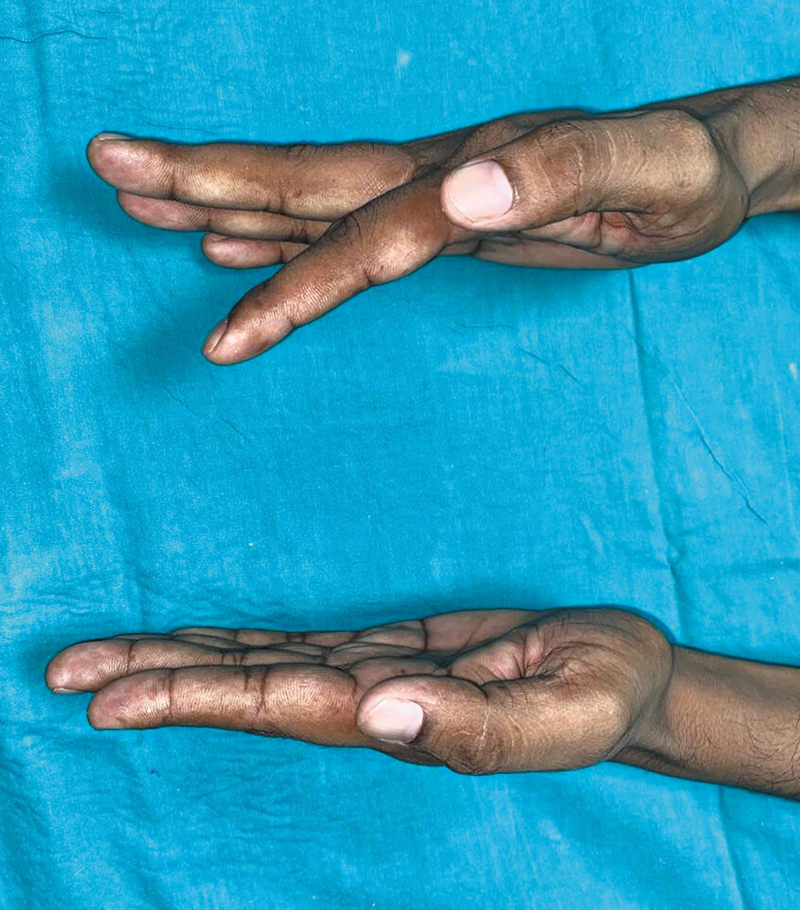
Preoperative picture showing inability to extend right index finger.

**Fig. 2:**
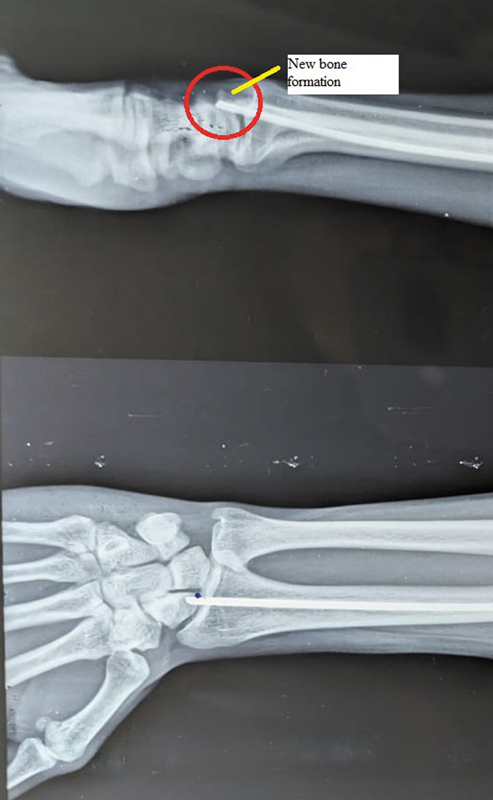
Preoperative radiographs of the right hand and distal forearm showing intramedullary titanium elastic nail system (TENS) nail impinging the extensor compartment.

On examination, the patient had complete loss of active extension of his right index finger at the metacarpophalangeal (MCP) joint, both independently and along with EDC of other fingers ( Fig. 1 ). The extension of other fingers and the thumb was preserved. Ultrasonography (USG) scan of hand showed complete discontinuity of both EDC and EIP tendons of the index finger, with a 6-cm gap between the proximal and distal cut ends. Proximal ends of the cut tendons were 3.7 and 4 cm proximal to wrist joint while the distal ends were 2 cm distal to second CMC joint. Radiographs revealed an intramedullary TENS nail in the radius, with the dorsal tip of the nail abutting the extensor compartment ( Fig. 2 ).

## Surgical Management

**Fig. 3:**
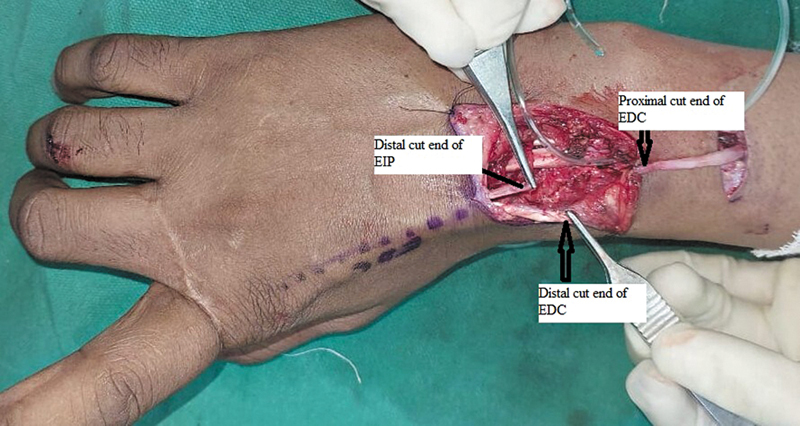
Intraoperative photo showing cut ends of extensor indicis proprius (EIP) and extensor digitorum communis (EDC) tendons.

**Fig. 4:**
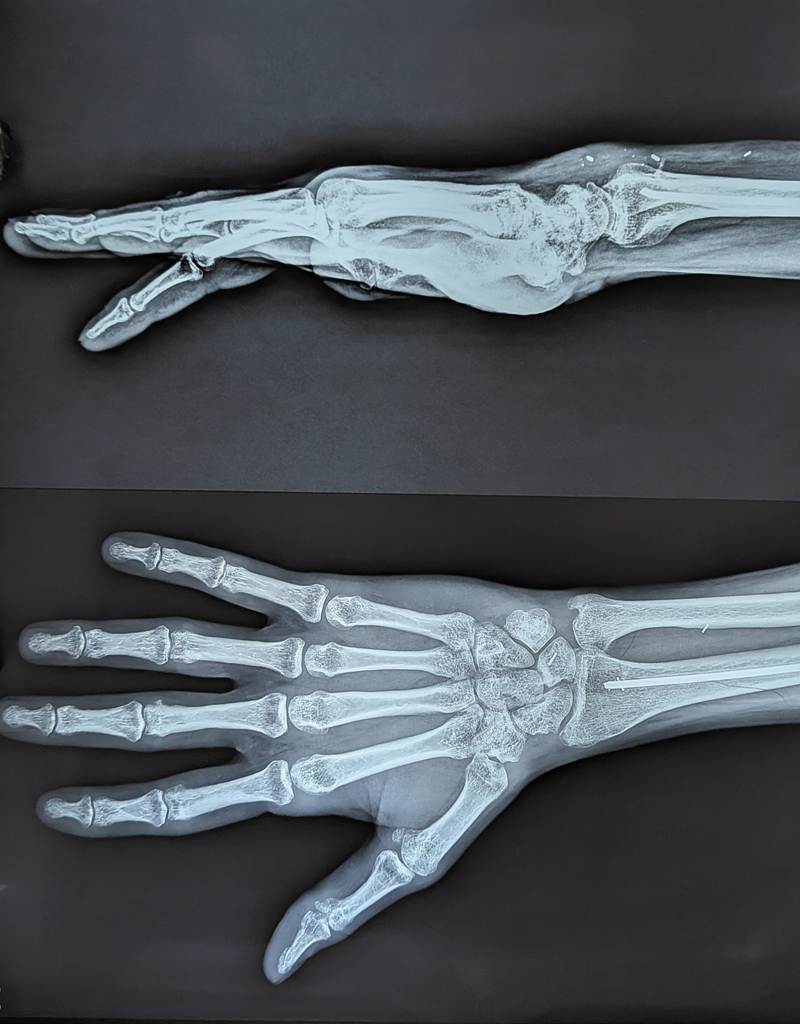
Postoperative radiograph of the right hand and distal forearm showing the shortened intramedullary nail no longer impinging the extensor compartment.

Under regional anesthesia, a “Z” incision on dorsal wrist and distal forearm was made. Extensor retinaculum was partially opened. Both EDC and EIP tendons of the index finger were found to be ruptured. The cut ends were frayed and located approximately 6 cm apart ( Fig. 3 ). EPL tendon was intact. At the entry point of the TENS nail in the distal radius, new bone had formed. The new bone was removed by burring, but the nail could not be removed. The protruding end of the nail was cut short, and the remaining nail was left inside the medullary canal ( Fig. 4 ). The nail was unlikely to migrate due to bony overgrowth and distal anchorage.

**Fig. 5:**
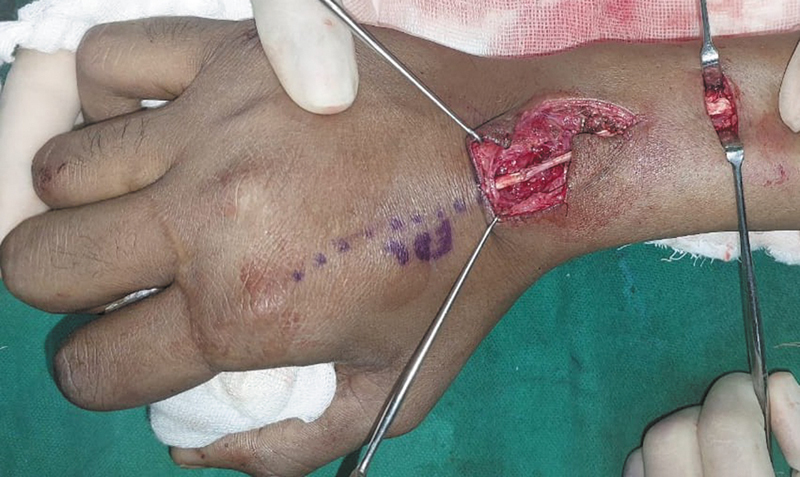
Intraoperative photo showing extensor digitorum communis (EDC) index finger tendon reconstruction using palmaris longus graft.

Primary tendon grafting was performed using palmaris longus tendon graft to reconstruct the EDC tendon of the index finger. The distal tendon juncture was achieved using modified Kessler's technique and the proximal end was secured using a Pulvertaft weave technique ( Fig. 5 ). The EIP tendon was noted to be very thin; therefore, it was not reconstructed.

Postoperatively, the hand was immobilized with wrist in 30 degrees extension and index finger MP joint in 10 to 20 degrees flexion with IP joints straight using a volar splint for 3 weeks. Early active supervised physiotherapy was started thereafter. At 6 weeks follow-up, the patient could make a fist and regained useful range of index finger extension.

## Discussion

While EPL tendon ruptures are well documented after distal radius fractures or its fixation, literature on isolated EDC/EIP attrition ruptures is limited. We are unaware of any cases of isolated EDC/EIP attrition ruptures related specifically to intramedullary TENS nailing of distal radius fracture.

Rupture of EDC and EIP tendons along with EPL has been reported. McKinney et al reported a case of delayed EIP and EDC tendons of index finger along with EPL rupture after a conservatively managed distal radius fracture at 14 weeks after injury. 1 Nguyen et al reported delayed rupture of EIP and EPL following volar plating of distal radius fracture. 2 EIP and EPL were found to be ruptured in their case due to penetration of screws into the third and fourth extensor compartment. Bhardwaj et al reported two cases of concomitant rupture of EPL and index extensor tendons following distal radius plating. 3

Although attrition rupture of extensor tendons of index finger due to old fracture of third metacarpal bone or carpal bone is reported, rupture of these tendons after distal radius fracture is rarely reported. On further literature search, report of three cases of isolated rupture of the extensor tendons of the index finger after *closed* reduction of the distal radius fracture were found. Bhardwaj et al reported a case of EIP rupture out of 21 patients with distal radius fracture. 4 Ghijselings et al reported an ED tendon tear in a 12-year-old girl 12 weeks after closed reduction of a distal radius fracture. The tear was attributed to abrasion of the tendon against a small flake of dorsally tilted cortical bone not seen on post-reduction X-rays. 5 Piotuch et al reported a case of a 17-year-old with delayed EIP tendon rupture following entrapment of the tendon in the bony callus after a distal radial fracture. 6 Furuya et al described a risk of extensor tendon entrapment associated with closed reduction of distal radius fracture with palmar displacement in a case report of a 16-year-old's EDC tear of index finger. 7 Rupture of these tendons after *open* reduction and internal fixation of distal radius fracture using volar plates and screws is reported by few authors. Hattori et al presented a case of delayed rupture of EDC tendon 7 years after volar plating of distal radius fracture. Previously reported cases as mentioned earlier resulted from the implants used in older days. Nowadays TENS is preferred over plating for osteosynthesis of forearm bones. Rupture of these tendons after intramedullary nailing, as observed in our case, is not reported. 8

In our case, the cause was chronic attrition due to contact of the extensor tendons of index finger with newly formed bone around the retained nail.

## Management

**Video 1** Postoperative recovery of extension of index finger.


Diagnosis of extensor tendon rupture can be confirmed on clinical examination and with appropriate imaging. Surgical exploration is essential as loss of index finger extension is functionally very disabling. Reconstructive options include direct repair (rarely feasible in chronic cases) or tendon transfer (e.g., cut distal end of EIP to adjacent EDC or flexor digitorum superficialis [FDS] middle finger transfer) or tendon grafting using palmaris longus or plantaris tendons.

In this case, cut distal end of EIP adjacent to EDC could have been performed. But we preferred anatomical reconstruction using palmaris longus tendon grafting as it provided independent index finger extension. Anatomical reconstruction in a young patient with requirement of strong index finger function for his job was more optimal treatment than tendon transfer or end-to-side suturing of extensor tendons. Also, the patient was 3 months post-rupture. Flexor digitorum superficialis (FDS) middle finger tendon transfer would have further delayed rehabilitation due to the time required for the strengthening of the donor tendon. We could start supervised physiotherapy from the 3rd week, which we believe was much easier for the patient than biofeedback training/re-education after tendon transfer. Early active supervised motion protocols after 3 weeks of immobilization helped in excellent functional recovery ( Video 1 ).

This case reminds us to consider the potential complication of tendon rupture due to any hardware used in patients with distal radius fracture, even years after surgery and the need to assess donor tendons before proceeding with reconstruction.

## Conclusion

Delayed rupture of extensor tendons of hand can occur due to a variety of reasons. Post-traumatic attrition rupture of EPL tendon following distal radius fractures is well known. Isolated rupture of both the EDC and EIP tendons of index finger without EPL rupture can occur but is very rare. High index of clinical suspicion coupled with necessary imaging followed by reconstruction using tendon graft can yield excellent functional recovery. Patients who have undergone distal radius fracture fixation should be counseled regarding retained hardware and its prophylactic removal to prevent future tendon rupture.
